# Mapping of a Mycoplasma-Neutralizing Epitope on the Mycoplasmal p37 Protein

**DOI:** 10.1371/journal.pone.0169091

**Published:** 2016-12-30

**Authors:** Min Kyu Kim, Won-Tae Kim, Hyun Min Lee, Hong Seo Choi, Yu Ra Jo, Yangsoon Lee, Jaemin Jeong, Dongho Choi, Hee Jin Chang, Dae Shick Kim, Young-Joo Jang, Chun Jeih Ryu

**Affiliations:** 1 Institute of Anticancer Medicine Development, Department of Integrative Bioscience and Biotechnology, Sejong University, Seoul, Korea; 2 Department of Laboratory Medicine, Hanyang University College of Medicine, Seoul, Korea; 3 Department of Surgery, Hanyang University College of Medicine, Seoul, Korea; 4 Center for Colorectal Cancer, Research Institute and Hospital of National Cancer Center, Goyang-si, Korea; 5 Department of Pathology, Samsung Medical Center, Sungkyunkwan University School of Medicine, Seoul, Korea; 6 Department of Nanobiomedical Science, BK21 PLUS Global Research Center for Regenerative Medicine, Dankook University, Cheonan, Korea; Universidade Federal de Pelotas, BRAZIL

## Abstract

Many studies have shown that the mycoplasmal membrane protein p37 enhances cancer cell migration, invasion, and metastasis. Previously, we generated 6 monoclonal antibodies (MAbs) against the mycoplasmal protein p37 and showed the presence of mycoplasma-infected circulating tumor cells in the blood of hepatocellular carcinoma patients by using CA27, one of the six MAbs. When mycoplasmas were incubated with cancer cells in the presence of CA27, mycoplasma infection was completely inhibited, suggesting that CA27 is a neutralizing antibody inhibiting mycoplasma infection. To examine the neutralizing epitope of CA27, we generated a series of glutathione S-transferase (GST)-fused p37 deletion mutant proteins in which p37 was partly deleted. To express p37-coding sequences in *E*.*coli*, mycoplasmal TGA codons were substituted with TGG in the p37 deletion mutant genes. GST-fused p37 deletion mutant proteins were then screened to identify the epitope targeted by CA27. Western blots showed that CA27 bound to the residues 216–246 on the middle part of the p37 protein while it did not bind to the residues 183–219 and 216–240. Fine mapping showed that CA27 was able to bind to the residues 226–246, but its binding activity was relatively weakened as compared to that to the residues 216–246, suggesting that the residues 226–246 is essential for optimal binding activity of CA27. Interestingly, the treatment of the purified GST-tagged epitopes with urea showed that CA27 binding to the epitope was sodium dodecyl sulfate-resistant but urea-sensitive. The same 226–246 residues were also recognized by two other anti-p37 MAbs, suggesting that the epitope is immunodominant. The identification of the novel neutralizing epitope may provide new insight into the interaction between the p37 protein and host receptors.

## Introduction

Mycoplasmas are the smallest bacteria capable of independent replication. They are tiny, wall-free prokaryotic organisms that live well, either attached to the eukaryotic plasma membrane or inside the eukaryotic cells. Because these bacteria survive well with eukaryotic cells without noticeable symptoms, mycoplasma infection is one of major concerns for animal and human cell cultures. *Mycoplasma hyorhinis* (*M*. *hyorhinis*)-encoded protein p37 consists of part of a high-affinity transport system in many mycoplasmas [1]. Originally, the mycoplasmal p37 protein was found to induce invasiveness in mouse sarcoma cells [2, 3]. Many studies have shown that persistent exposure to mycoplasmas is associated with oncogenic transformation in many human cancers [4–7]. Subsequent studies have demonstrated that the mycoplasmal p37 protein results in the proliferation, invasiveness, and metastases of cancer cells [8–10]. A recent study also demonstrated that the p37 protein expression predicts poor survival and metastasis in gastric cancer patients [11].

In the previous study, we generated 75 MAbs specific to mycoplasma-infected A549 lung cancer cells, and found that 6 MAbs recognized the p37 protein [12]. By using CA27 (IgG_3_, κ), one of the 6 MAbs, we showed the first evidence for the presence of mycoplasma-infected circulating tumor cells (CTCs) in the blood of hepatocellular carcinoma (HCC) patients [12]. Subsequent studies revealed that CA27 was able to inhibit the infection of mycoplasma to HCC cancer cells, indicating that the epitope of CA27 is a mycoplasma infection-neutralizing epitope on the p37 protein. To investigate the neutralizing epitope of CA27, we generated a series of deletion mutants of the p37 protein, expressed them in *E*. *coli* as glutathione S-transferase (GST)-fusion proteins, and found that the epitope of CA27 is within the residues 226–246 of the p37 protein. We also found that two other anti-p37 antibodies generated against the p37 protein bound to the same epitope, suggesting that the novel epitope is immunodominant. The results of this study show a novel neutralizing epitope of mycoplasmas, which provides new insight into the interaction between the p37 protein and host receptors.

## Materials and Methods

### Cell culture

Mycoplasma-free A549 (human lung adenocarcinoma cell line) and Huh7 (human liver carcinoma cell line) cells were purchased from the Korean Cell Line Bank and maintained in RPMI-1640 medium supplemented with 10% fetal bovine serum (Biowest, Riverside, MO, USA) and antibiotic-antimycotic solution (Welgene, Seoul, Korea).

### Mycoplasma culture

Mycoplasma-free Huh7 cells were infected with mycoplasmas originated from mycoplasma-infected A549 cells [12]. Infected Huh7 cells were serially passaged, harvested, and stored at -80°C. Titer of mycoplasmas was performed using Mycoplasma IST2 kit (bioMérieux, Marcy-l'Etoile, France) and blood agar plates. According to the manufacturer's instructions of Mycoplasma IST2 kit (bioMérieux), the inoculated strip was incubated at 35°C in a CO_2_ incubator and observed for color changes, and the results were interpreted after 24 and 48 hours of incubation. Additionally, 1 μl and 10 μl of the culture supernatants of infected Huh7 cells were spread on blood agars (Asan Pharmaceutical, Hwaseong, Korea), respectively and incubated using GasPak EZ anaerobe pouch system (BD Diagnostics, Sparks, MD, USA) at 35°C for 96 hours.

### Antibody-mediated neutralization of mycoplasmas

RPMI-1640 media containing mycoplasmas (1x10^5^ CFU/ml) were pre-incubated with boiled CA27 (3 μg/ml) and increasing concentrations (1–3 μg/ml) of CA27 at 37°C for 3 hours, added to mycoplasma-free Huh7 cells (2 x 10^5^ cells/well) in 12 well plates, and further incubated at 37°C for 2 days in a 5% CO_2_ incubator. After washing three times with phosphate buffered saline (PBS, pH7.4), cells were detached by 0.25% Trypsin-EDTA (Welgene). The cells (1×10^4^ cells) were washed with PBA (PBS plus 0.1% bovine serum albumin) and analyzed by flow-cytometric analysis with CA27 followed by a further incubation with fluorescein isothiocyanate (FITC)-conjugated mouse IgG antibody (Santa Cruz Biotechnology, Santa Cruz, CA, USA) for 30 min at room temperature (RT). CA27 binding to mycoplasma-infected cells was determined by flow cytometry and shown as mean fluorescence intensity (MFI). To detect mycoplasmas by polymerase chain reaction (PCR) technique, infected Huh7 cells (2 x 10^5^ cells) were suspended in distilled water after harvest. Suspended cells were boiled at 100°C, and the supernatants were then subjected to PCR using e-Myco™ VALid-Q mycoplasma qPCR detection kit (Intron, Seoul, Korea). The amplified PCR products were visualized by agarose gel electrophoresis analysis.

### Preparation and induction of GST-fusion protein

Serially truncated mycoplasmal p37 proteins were expressed as fusion proteins with GST proteins, as described previously [13]. The coding sequences of serially truncated p37 genes were synthesized by PCR from mycoplasma-infected A549 cells using various 5’-primer and 3’-primers and subcloned into the EcoRI/SalI sites of pGEX4T-2 (GE Healthcare, Seoul, Korea) to yield the expression plasmids. All primer sequences are listed in **Table 1**. TGA is not a termination codon, but codes for tryptophan in *M*. *hyorhinis*. Therefore, mycoplasmal TGA codons were modified to TGG, a universal codon for tryptophan, using mutagenic oligonucleotide primers. Each expression plasmid was confirmed by DNA sequencing, and introduced into *E*. *coli* DH5α cells to express the GST-p37 fusion proteins. The expression of the fusion proteins was induced by 0.1 mM isoprophyl-β-D-thiogalactopyranoside (IPTG) at 32°C for 6 hours. The induced bacterial cells were washed with pre-chilled PBS (pH 7.4), incubated with acetone on ice for 5 min, and lysed in 1% sodium dodecyl sulfate (SDS) supplemented with 100 μg/ml phenylmethanesulfonyl fluoride (PMSF) for 2 min at RT. Cell lysates were clarified by centrifugation and the protein concentration of the samples was measured by bicinchoninic assay (Thermos Scientific, Seoul, Korea). The cell lysates were subjected to 12.5% SDS-polyacrylamide gel electrophoresis (PAGE), stained with Coomassie Brilliant Blue R-250, and analyzed by Western blot analysis as described, previously [14, 15]. To do Western blots with the purified GST-p37 fusion proteins, the recombinant proteins were also purified by affinity chromatography on glutathione agarose beads according to the protocol provided by the supplier (GE Healthcare).

**Table 1:** Primer sequences for the generation of truncated mutants of the p37 protein.

Residues	Position	Sequence
42–190	N-terminus	CCG AAT TCC CGA TAA AAG TAT AAC AT
	C-terminus	CCG TCG ACT CAA CTT GCA TAT GGA G
183–246	N-terminus	CCG AAT TCC CGT TGA AAC TCC ATA TGC AAG TTG GAC TGA TGA AAA TCA TAA GTG GAA TGG TAA TGT T
	N-terminus	ATG ATT TGG ATA AAA GGT AAT GAT
	C-terminus	TTT TAT CCA AAT CAT TCC TCT ATA
	C-terminus	GGG TCG ACAA AAT TTC TAA ATG TAT TCC AAT CTT TAT CAT TCC AAG CTT TTT TAA TTT T
241–313	N-terminus	CCG AAT TCC CAA TAC ATT TAG AAA TTT TG
	C-terminus	CCG TCG ACT CAA GCA AAA GAA CCT TCT
315–403	N-terminus	CCG AAT TCC CAC ACA TAA CAA ATC AGC A
	C-terminus	CCG TCG ACA ATT TAT TTA ATG GCT TTT TC
183–219	N-terminus	CCG AAT TCC CGT TGA AAC TCC ATA TGC AAG TTG GAC TGA TGA AAA TCA TAA GTG GAA TGG TAA TGT T
	C-terminus	GGG TCG ACT CAA ATC ATT CCT CTA TAA AA
216–246	N-terminus	CCG AAT TCC CTA TAG AGG AAT GAT TTG GAT AAA AGG TAA TGA T
	C-terminus	GGG TCG ACA AAA TTT CTA AAT GTA TT CCA ATC TTT ATC ATT CCA AGC TTT TTT AAT TTT
226–246	N-terminus	CCG AAT TCC CGA AAC TCT AGC TAA AAT TAA AA
	C-terminus	GGG TCG ACA AAA TTT CTA AAT GTA TT CCA ATC TTT ATC ATT CCA AGC TTT TTT AAT TTT
216–240	N-terminus	CCG AAT TCG AGG AAT GAT TTG GAT T
	C-terminus	CCG TCG ACT AGA GTT TCA TCA TTA CC

### Western blot

Mycoplasma infected A549 cells were lysed in ice-cold immunoprecipitation buffer (150 mM NaCl, 1% NP 40, 0.5% deoxycholate, 0.1% SDS, 25 mM Tris-HCl, pH 7.5, 5 mM EDTA, 2 μg/ml aprotinin, 100 μg/ml PMSF, 5 μg/ml leupeptin, 1mM NaF and 1mM NaVO_3_) at 4°C for 30 min. Cell lysates and GST-p37 proteins were boiled for 10 min with SDS-PAGE sample buffer. Purified GST-p37 fusion proteins were also treated with 4M urea for 10 min at RT before the treatment of SDS sample buffer. The cell lysates and GST-p37 proteins were resolved by 12% SDS-PAGE and transferred to nitrocellulose membrane. Western blotting was performed, as described previously [14, 15]. The membrane was blocked by 5% skim milk in Tris-buffered saline with 0.1% Tween 20 at RT for 1 hour and incubated with CA27 and α-GST antibodies (Santa Cruz Biotechnology, Santa Cruz, CA, USA) for 1 hour. The membranes were incubated with anti-mouse immunoglobulin G (IgG)-horseradish peroxidase at RT for 1 hour. The proteins were visualized by enhanced chemiluminescence detection reagent (Advansta, Menlo Park, CA, USA).

### Hydrophobicity analysis

To obtain values that define relative hydrophobic character of amino acid residues, the predicted hydrophobicity profile of the p37 protein was obtained using ProtScale (http://www.expasy.ch/cgi-bin/protscale.pl).

## Results and Discussion

### CA27 neutralizes mycoplasma infection

**Fig 1:**
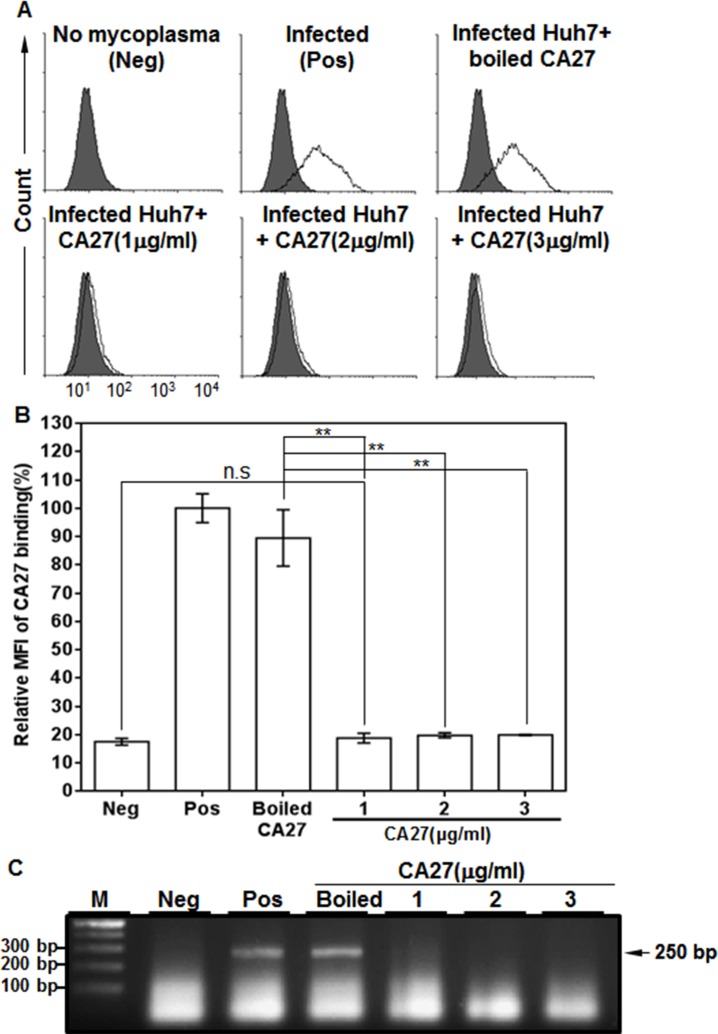
CA27 neutralizes mycoplasma infection. (A) Mycoplasmas (1x10^5^ CFU/ml) were incubated with PBS (positive control), boiled CA27 (negative control, 3 μg/ml), or active CA27 (1–3 μg/ml), added to Huh7 cells, and then cultured for 2 days. Mycoplasma infection of Huh7 cells was then measured by flow cytometric analysis with CA27. (B) Graphic presentation of relative CA27 binding to mycoplasma-infected Huh7 cells. MFI (mean fluorescence intensity) values of CA27 binding were measured and presented as percentages. (C) Detection of mycoplasma DNA in mycoplasma-infected Huh7 cells by a commercial mycoplasma qPCR detection kit. Mycoplasma DNAs were detected in mycoplasma-free (neg), mycoplasma-infected (pos), and mycoplasma/ CA27-treated Huh7 cells. The arrow indicates the position of the amplified mycoplasmal DNA. The expected size of the PCR product is 250 base pairs. M represents the marker lane. The graph represents the mean values of three independent determinations ± standard deviation. Statistical analysis used Student’s t‐test (n.s, not significant, * *p*<0.05, ** *p*<0.01).

The mycoplasma p37 protein is localized on the surface of mammalian cells after infection [3]. A polyclonal anti-p37 antibody blocks the infection of *M*. *hyorhinis* to gastric cancer cells, GES-1, and HUVEC cells, indicating that p37 is essential for *M*. *hyorhinis* infection [11]. By using CA27, we detected CTCs in the peripheral circulating blood of patients with HCC, which is the first evidence for the presence of mycoplasma-infected CTCs in cancer patients [12]. In order to examine whether CA27 was able to block the infection of mycoplasmas to HCC cells, mycoplasmas (1x10^5^ CFU/ml) were incubated with CA27 and added to Huh7 cells. Mycoplasma infection was then measured by flow cytometric analysis with CA27. Flow cytometric analysis showed CA27 binding in mycoplasma-infected Huh7 cells (**Fig 1A and 1B**). When mycoplasmas were incubated with CA27 or boiled CA27, mycoplasma infection was inhibited in CA27-treated Huh7 cells while it was not inhibited in boiled CA27-treated Huh7 cells (**Fig 1A and 1B**). To further confirm whether CA27 blocks the infection of mycoplasmas to Huh7 cells, cells were detached, boiled, and subjected to qPCR analysis using mycoplasma-specific PCR primers. As expected, mycoplasmas were not detected in CA27-treated Huh7 cells while they were detected in boiled CA27-treated Huh7 cells (**Fig 1C**). Taken together, the results suggest that CA27 is able to neutralize the infection of mycoplasmas to Huh7 cells, and the epitope of CA27 is a mycoplasma infection-inhibiting epitope on the p37 protein.

### Expression of GST-p37 fusion proteins and epitope mapping of CA27

**Fig 2:**
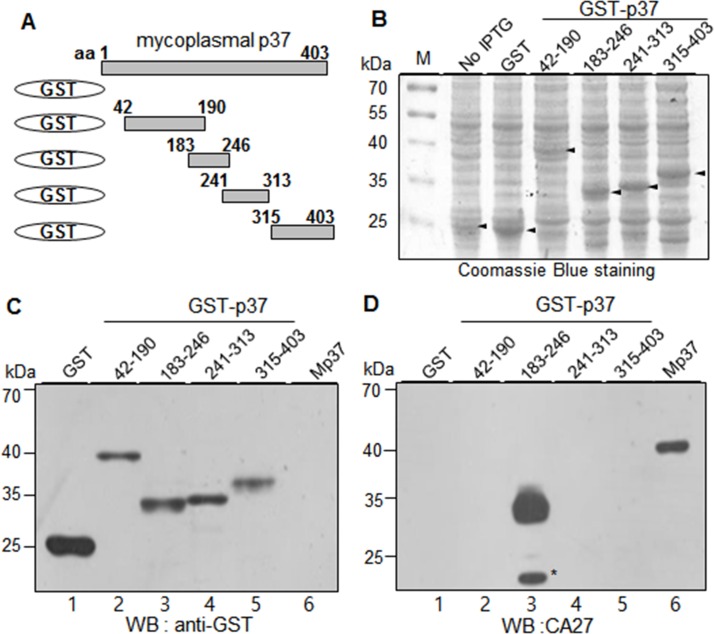
CA27 recognizes the residues 183–246 of the mycoplasmal p37 protein expressed as GST-fusion proteins. (A) Schematic diagram of recombinant p37 fragments (residues 42–190, 183–246, 241–313, and 315–403). (B) Individual fusion proteins were expressed in *E*. *coli* as fusion proteins with GST tag at the N-terminus and stained with Coomassie Brilliant Blue R250 after SDS-PAGE. (C-D) Western blot analyses of GST-P37 fusion proteins with α-GST (C) and CA27 antibodies (D). Mp37 represents the mycoplasmal p37 protein from the extract of mycoplasma-infected cancer cells. The asterisks indicate partial degradation of GST-p37 fusion proteins.

CA27 recognizes the p37 protein in the Western blot analysis, suggesting that CA27 binds to a linear epitope on the p37 protein [12]. To determine the epitope of CA27, a series of truncated mutants of the p37 gene were synthesized by PCR and fused to GST gene to construct a series of expression plasmids (**Fig 2A**). Five TGA codons located in the middle part of the p37 gene (residues 183–246) codes for tryptophan in mycoplasmas. Therefore, the TGA codons were mutated into universal codon TGG using mutagenic oligonucleotide primers (**Table 1**). GST-p37 fusion proteins that included the residues 42–190, 183–246, 241–313, or 315–403 of the p37 protein were constructed and expressed in *E*.*coli* after IPTG induction. The fragmentation of the p37 protein was designed to cover all hydrophilic regions of the p37 protein (**S1 Fig**). The hydrophobicity profile of the p37 protein was obtained according to the method described previously [16]. The protein extracts of the recombinant cells were analyzed by SDS-PAGE, visualized by Coomassie Blue staining, and analyzed by Western blot analysis with an anti-GST antibody (α-GST) (**Fig 2B and 2C**). The cell lysate from mycoplasma-infected cancer cells was also included in the analysis as a control [12]. All of the GST-p37 fusion proteins were readily expressed and detected (**Fig 2C**). The same lysates were then subjected to Western blot analysis with CA27 (**Fig 2D**). As a control, CA27 recognized the full-length form of the p37 protein from mycoplasma-infected cancer cells **(Fig 2D** lane 6). CA27 specifically recognized one truncated mutant form containing the residues 183–246 of the p37 protein, while it did not recognize the other truncated mutant forms (residues 42–190, 241–313, and 315–403) (**Fig 2C and 2D**). The partially degraded form of the truncated mutant form containing the residues 183–246 was also detected below the main protein band. The results suggest the epitope of CA27 is located within the residues 183–246 of the p37 protein.

### CA27 recognizes the residues 226–246 of the p37, and the binding is SDS-resistant but urea-sensitive

**Fig 3:**
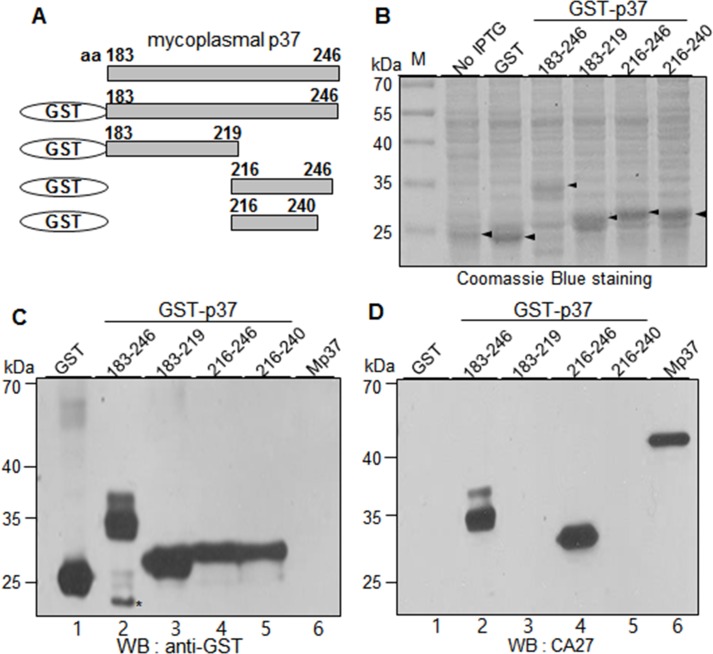
CA27 recognizes the residues 216–246 of the p37 protein. (A) Schematic diagram of recombinant p37 fragments (residues 183–219, 216–246, and 216–240). (B) Individual fusion proteins were expressed in *E*. *coli* as fusion proteins with GST tag at the N-terminus and stained with Coomassie Brilliant Blue R250 after SDS-PAGE. (C-D) Western blot analyses of GST-P37 fusion proteins with α-GST (C) and CA27 antibodies (D). Mp37 represents the mycoplasmal p37 protein from the extract of mycoplasma-infected cancer cells. The asterisks indicate partial degradation of GST-p37 fusion proteins.

To dissect the epitope of CA27, we generated and expressed GST-p37 fusion proteins that included the residues183-246, 183–219, 216–246, or 216–240 of the p37 protein. As shown in **Fig 3A and 3B**, the GST-p37 fusion proteins were readily expressed and detected. CA27 did not bind to the residues 183–219 while it still bound to the residues 183–246 (**Fig 3D** lanes 2 and 3). When the binding activity of CA27 was compared between the residues 216–246 and 216–240, CA27 did not show the binding activity to the residues 216–240 while it showed the binding activity to the residues 216–246 (**Fig 3D** lanes 4 and 5). The result suggests that the residues 241–246 are necessary for the binding activity of CA27. However, the residues 216–219 and 241–246 were not enough to retain the binding activity of CA27 because CA27 was not able to recognize the residues 183–219 and 241–313 (**Fig 3D** lane 3 and **Fig 2D** lane 4).

**Fig 4:**
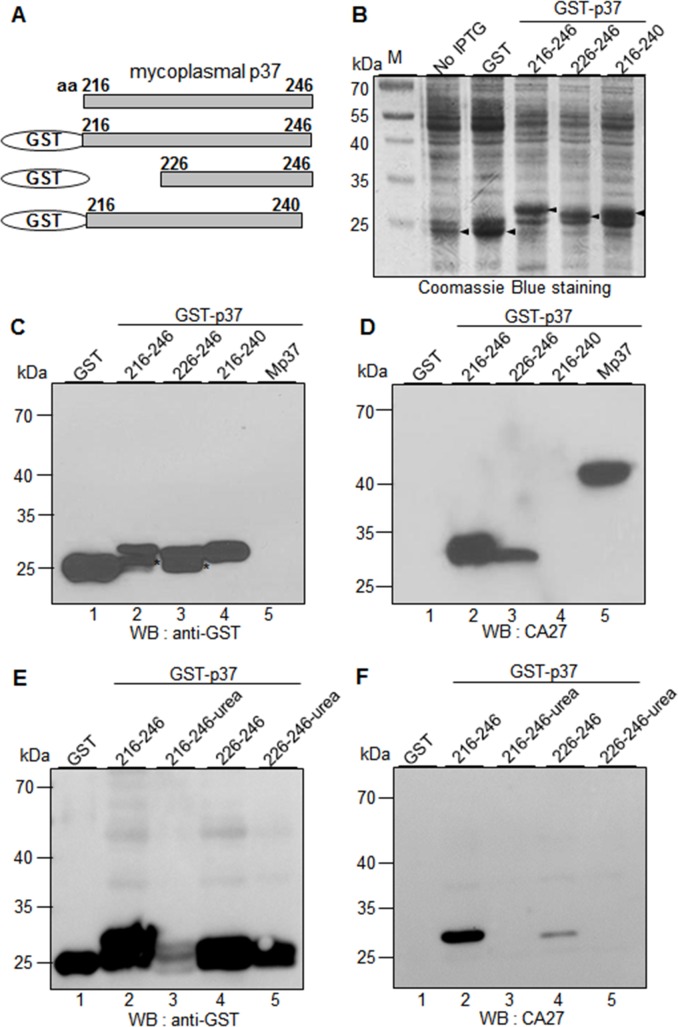
CA27 recognizes the residues 226–246 of the p37 protein, and the binding is SDS-resistant but urea-sensitive. (A) Schematic diagram of recombinant p37 fragments (residues 216–246, 226–246, and 216–240). (B) Individual fusion proteins were expressed in *E*. *coli* as fusion proteins with GST tag at the N-terminus and stained with Coomassie Brilliant Blue R250 after SDS-PAGE. (C-D) Western blot analyses of GST-p37 fusion proteins with α-GST (C) and CA27 antibodies (D). Mp37 represents the mycoplasmal p37 protein from the extract of mycoplasma-infected cancer cells. The asterisks indicate partial degradation of GST-p37 fusion proteins. (E-F) Western blot analysis of the purified GST-p37 fusion proteins in the presence or absence of urea with α-GST (E) and CA27 antibodies (F).

To further define the epitope of CA27, we constructed and expressed GST-p37 fusion protein that included the residues 226–246 of the p37 protein. Among the residues 216–246, 226–246, and 216–240, CA27 showed the binding activities to the residues 216–246 and 226–246. However, the relative binding activity to the residues 226–246 was weakened as compared to that to the residues 216–246 **(Fig 4C and 4D** lanes 2 and 3). Thus, the residues 226–246 are essential for optimal binding activity of CA27.

To examine whether CA27 binding to the residues 226–246 depends on the locally folded structure of the epitope, GST, GST-p37 (residues 216–246), and GST-p37 (residues 226–246) proteins were purified (data not shown) and treated with urea before the treatment of SDS sample buffer. Urea treatment inhibited α-GST binding to the GST-p37 (residues 216–246) but not to the GST-p37 (residues 226–246) in the Western blot analysis (**Fig 4E**). As expected, CA27 bound to both the residues 216–246 and 226–246 in the absence of urea, but urea treatment abrogated CA27 binding to both GST-p37 proteins (**Fig 4F**). Thus, CA27 binding to the epitope (the residues 226–246) was SDS-resistant but urea-sensitive, suggesting that CA27 may recognizes the locally folded structure of the residues 226–246 of the p37 protein.

### CA63 and CA279 also recognize the resides 226–246 and neutralize mycoplasma infection

**Fig 5:**
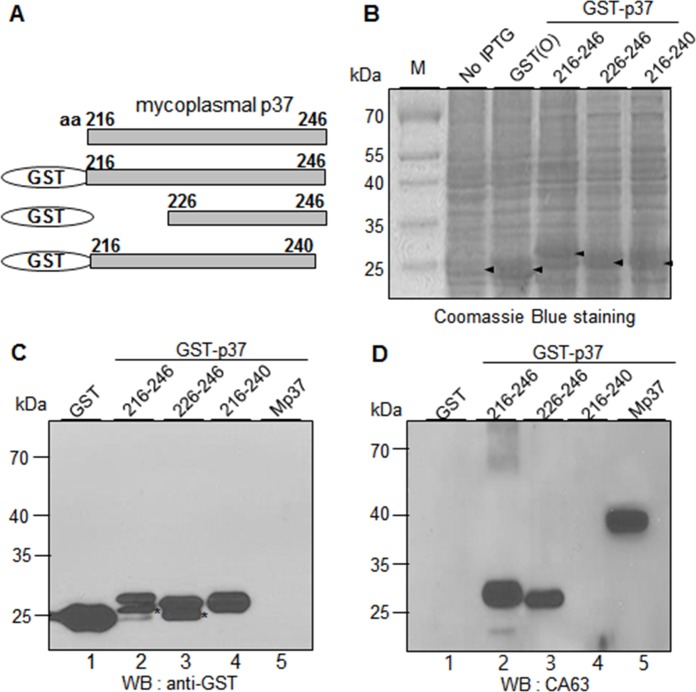
CA63 recognizes the residues 226–246 of the p37 protein. (A) Schematic diagram of recombinant p37 fragments (residues 216–246, 226–246, and 216–240). (B) Individual fusion proteins were expressed in *E*. *coli* as fusion proteins with GST tag at the N-terminus and stained with Coomassie Brilliant Blue R250 after SDS-PAGE. (C-D) Western blot analyses of GST-p37 fusion proteins with α-GST (C) and CA27 antibodies (D). Mp37 represents the mycoplasmal p37 protein from the extract of mycoplasma-infected cancer cells. The asterisks indicate partial degradation of GST-p37 fusion proteins.

**Fig 6:**
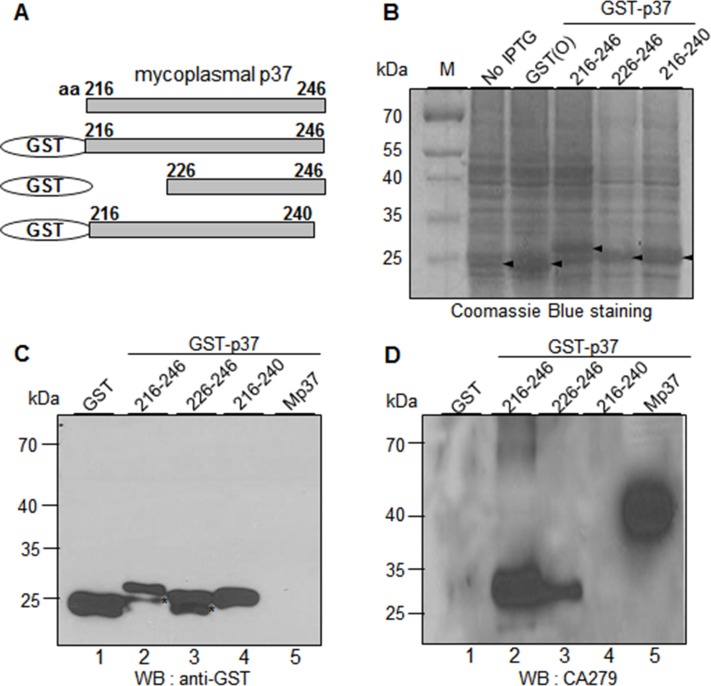
CA279 recognizes the residues 226–246 of the p37 protein. (A) Schematic diagram of recombinant p37 fragments (residues 216–246, 226–246, and 216–240). (B) Individual fusion proteins were expressed in *E*. *coli* as fusion proteins with GST tag at the N-terminus and stained with Coomassie Brilliant Blue R250 after SDS-PAGE. (C-D) Western blot analyses of GST-p37 fusion proteins with α-GST (C) and CA27 antibodies (D). Mp37 represents the mycoplasmal p37 protein from the extract of mycoplasma-infected cancer cells. The asterisks indicate partial degradation of GST-p37 fusion proteins.

In the previous study, we produced 75 MAbs against mycoplasma-infected A549 cells [12]. Interestingly, 6 MAbs were able to immunoprecipitate the p37 protein as CA27 did (**S2 Fig, Table 2**). However, CA30 and CA193 were not able to recognize the p37 protein in the Western blot analysis, suggesting that they may recognize the p37 protein in a conformation-dependent manner. In order to define the epitopes of CA63 and CA279, the above GST-p37 fusion proteins were subjected to Western blot analysis with CA63 and CA279. CA63 and CA279 specifically recognized one truncated mutant form containing the residues 183–246 among residues 42–190, 241–313, 183–246, and 315–403 of the p37 protein (data not shown). In the fine epitope mapping, CA63 and CA279 showed the binding activities to the residues 216–246 and 226–246 among the residues 216–246, 226–246, and 216–240 as CA27 (**Figs 5 and 6**). Again, the relative binding activities of CA63 and CA279 to the residues 226–246 were relatively weakened as compared to that to the residues 216–246 (**Fig 5C and 5D** lanes 2 and 3; **Fig 6C and 6D** lanes 2 and 3). The results suggest that the residues 226–246 are also essential for optimal binding activities of CA63 and CA279.

**Table 2:** List of 6 MAbs against the mycoplasma p37 protein.

Name	Isotype	Reactivity to p37		Epitope
		Immunoprecipitation	Western blot	
CA27	IgG3, κ	Yes	Yes	226–246
CA30	IgG2a, κ	Yes	No	ND
CA63	IgG2b, κ	Yes	Yes	226–246
CA193	IgG1, κ	Yes	No	ND
CA149	IgG1, κ	Yes	ND*	ND
CA279	IgG2b, κ	Yes	Yes	226–246

*ND: not determined

**Fig 7:**
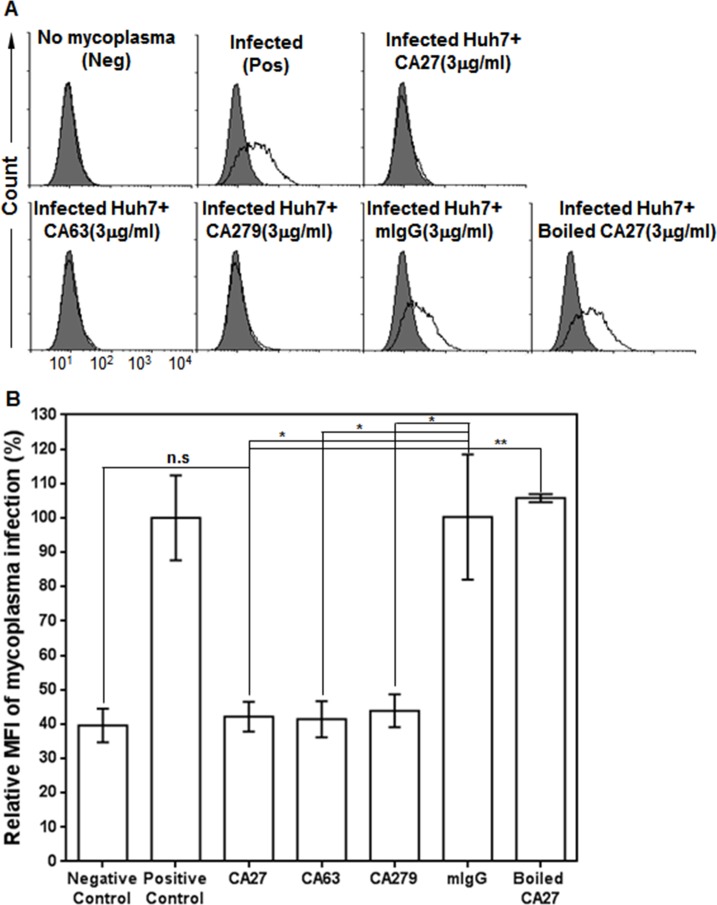
CA27, CA63, and CA279 neutralize mycoplasma infection with similar efficiency. (A) Mycoplasmas (1x10^5^ CFU/ml) were incubated with PBS (positive control), mIgG (negative control), boiled CA27, active CA27, CA63, or CA279, added to Huh7 cells, and then cultured for 2 days. Mycoplasma infection of Huh7 cells was then measured by flow cytometric analysis with CA27, CA63, or CA279. (B) Graphic presentation of relative CA27, CA63, and CA279 binding to mycoplasma-infected Huh7 cells. MFI (mean fluorescence intensity) values of CA27, CA63, and CA279 binding were measured and presented as percentages. The graph represents the mean values of three independent determinations ± standard deviation. Statistical analysis used Student’s t‐test (n.s, not significant, * *p*<0.05, **p<0.01).

In order to examine whether CA63 and CA279 were also able to block the infection of mycoplasmas to Huh7 cells, mycoplasmas were incubated with CA27, CA63, and CA279, and added to Huh7 cells. Mycoplasma infections were then measured by flow cytometric analysis with the antibodies. As expected, mycoplasma infections were inhibited with almost similar efficiency in CA27-, CA63-, and CA279-treated Huh7 cells, whereas mycoplasma infections were not inhibited in boiled CA27- and mouse IgG-treated Huh7 cells (**Fig 7A and 7B**). The results suggest again that CA27, CA63, and CA279 are able to neutralize the infection of mycoplasmas to Huh7 cells, and the epitope of those antibodies is a mycoplasma infection-inhibiting epitope on the p37 protein.

Most of antibody epitopes are in the range of 5–22 amino acid length, although they vary from 3 to 85 amino acids [17]. Therefore, it is possible to speculate that the real length of CA27 epitope may be smaller than that of the residues 226–246. However, there were critical residues for CA27 binding in the C-terminal residues 241–246. When the N-terminal residues 216–225 were deleted, the binding activity of CA27 was relatively weakened (**Fig 4D** lanes 2 and 3). Thus, the residues 226–246 seem to be an essential epitope for CA27 binding. The crystal structure of the p37 protein shows that the residues 225–245 of the p37 protein are in an alpha-helical configuration and exposed on the surface of domain II while the residues 217–223 are in a beta-strand configuration and located under the alpha-helix (Available from http://www.rcsb.org/pdb/explore/explore.do?structureId=3e78) [1, 18]. Therefore, we expected that the residues 216–225 were not necessary for CA27 binding because this part was located inside the overall structure in native p37 structure. As expected, CA27 retained the binding activity to the residues 226–246. However, CA27 binding to the residues 226–246 was weakened without the residues 216–225, suggesting that some residues in the residues 216–225 may be also associated with CA27 binding. Therefore, our results suggest that the core epitope of CA27 appears to be contained within residues 226–246 and that flanking sequences may either provide additional contact residues, or contribute to an overall conformation that cannot be achieved in their absence. When the purified GST-p37 (residues 226–246) was treated with urea, interestingly, CA27 binding was abrogated in the Western blot analysis (**Fig 4F**). The result suggests that CA27 may recognize the SDS-resistant and urea-sensitive local structure on the residues 226–246 of the p37 protein.

Previous studies found that mouse sarcoma FS9 cells become highly invasive with *M*. *hyorhinis* infection, and antibodies against the mycoplasmal p37 protein inhibit the invasiveness [19, 20]. Since then, many studies have shown that the mycoplasmal p37 protein increases the invasiveness and metastasis of cancer cells [8–10]. Addition of p37 to gastric carcinoma and prostate cancer cells increases the migration of the cancer cells [8, 10, 11]. The p37 protein also increases gene expression in fibroblasts associated with inflammation and cancer [21]. Thus, the p37 protein might play an important role in cancer progression. Using PD4 antibody targeting the N terminus of p37, the p37 protein was identified in many cancer tissues, including gastric, colon, esophageal, lung, breast, and glioma carcinomas [6]. During mycoplasma infections, the N-terminal region of the p37 protein binds to host annexin A2 and promotes NF-κB-dependent migration of human gastric cancer cells [11]. Another study shows that the C-terminal 20 amino acids of the p37 protein may be involved in p37-induced gene expression in NIH3T3 fibroblasts through toll-like receptor 4 signaling [21]. It is also possible to speculate that other parts of the p37 protein interact with unknown host receptors [21]. Thus, the interaction between the p37 protein and host receptors is still poorly understood. In this regard, CA27 could be a useful tool to study the interaction between host receptors and the p37 protein because it’s epitope is a novel linear epitope on the middle part of the p37 protein. Contrary to the hydrophobic N-terminal part of the p37 protein, the CA27-reactive middle part of the p37 protein is composed of hydrophilic amino acids, which could be surface-exposed and immunogenic (**S1 Fig**) [18]. Furthermore, CA27 was able to prevent the infection of mycoplasmas to cancer cells (**Fig 1**), suggesting that CA27-reactive epitope is critical for mycoplasma binding to host cells. Furthermore, multiple sequence alignment of p37-like proteins also shows that the residues 232–246 of CA27 epitope are relatively well-conserved among species [1]. Therefore, this novel epitope will provide critical insight into the interaction between the p37 protein and host receptors.

We generated 6 MAbs against the p37 protein [12] and found that 3 of them recognized the same 226–246 residues (**Figs 4, 5 and 6**). The findings suggest that the residues 226–246 of the p37 protein are highly immunogenic *in vivo*. Antibodies against the p37 protein are detected in the serum of men with newly diagnosed prostate cancer [22]. The present study leads us to suggest that antibodies recognizing the residues 226–246 of the p37 protein will be immunodominant antibodies in the serum of human with mycoplasma-infected diseases. Actually, we were readily able to detect mycoplasma-infected CTCs in the peripheral blood in patients with HCC by using CA27 antibody [12]. Thus, CA27 recognizing the residues 226–246 of the mycoplasma p37 protein would benefit the development of new diagnostic arrays in mycoplasma-associated diseases. Further investigation is needed to verify this speculation.

## Supporting Information

S1 Fig The hydrophobicity profile of the mycoplasmal p37 protein. The hydrophobicity profile was obtained using ProtScale (http://www.expasy.ch/cgi-bin/protscale.pl), using a window length of 9. Hydrophobic regions are above the line, and hydrophilic regions are below. The black bars indicate the p37 DNA fragments subcloned, and the red bar indicates the epitope of CA27.
(TIF)

S2 Fig Immunoprecipitation of p37 from mycoplasma-infected cancer cells with anti-p37 MAbs. Mycoplasma-infected A549 cells were subjected to immunoprecipitation with 6 indicated antibodies. Immunoprecipitated molecules were analyzed by Western blot analysis with CA27. Mp37 represents the mycoplasmal p37 protein from the extract of mycoplasma-infected cancer cells. The arrow indicates the position of the p37 protein.
(TIF)
