# Correction: Mapping of a Mycoplasma-Neutralizing Epitope on the Mycoplasmal p37 Protein

**DOI:** 10.1371/journal.pone.0172487

**Published:** 2017-02-14

**Authors:** Min Kyu Kim, Won-Tae Kim, Hyun Min Lee, Hong Seo Choi, Yu Ra Jo, Yangsoon Lee, Jaemin Jeong, Dongho Choi, Hee Jin Chang, Dae Shick Kim, Young-Joo Jang, Chun Jeih Ryu

There is an error in the caption for Fig 5. Please see the complete, correct Fig 5 caption here.

**Fig 5:**
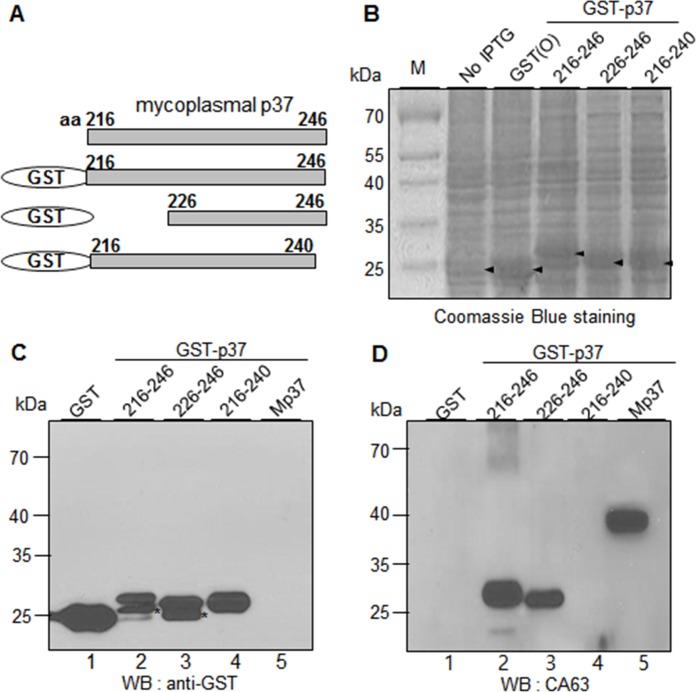
CA63 recognizes the residues 226–246 of the p37 protein. (A) Schematic diagram of recombinant p37 fragments (residues 216–246, 226–246, and 216–240). (B) Individual fusion proteins were expressed in *E*. *coli* as fusion proteins with GST tag at the N-terminus and stained with Coomassie Brilliant Blue R250 after SDS-PAGE. (C-D) Western blot analyses of GST-p37 fusion proteins with α-GST (C) and CA63 antibodies (D). Mp37 represents the mycoplasmal p37 protein from the extract of mycoplasma-infected cancer cells. The asterisks indicate partial degradation of GST-p37 fusion proteins.

There is an error in the caption for Fig 6. Please see the complete, correct Fig 6 caption here.

**Fig 6:**
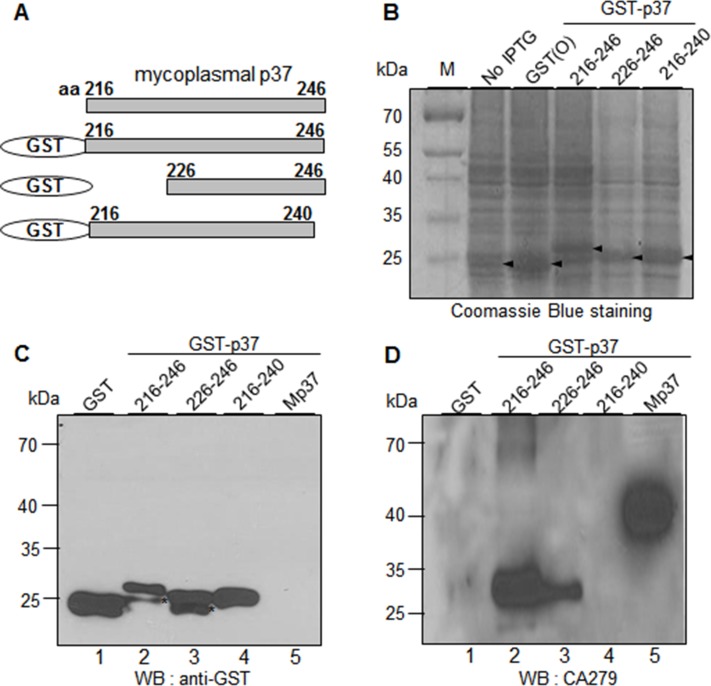
CA279 recognizes the residues 226–246 of the p37 protein. (A) Schematic diagram of recombinant p37 fragments (residues 216–246, 226–246, and 216–240). (B) Individual fusion proteins were expressed in *E*. *coli* as fusion proteins with GST tag at the N-terminus and stained with Coomassie Brilliant Blue R250 after SDS-PAGE. (C-D) Western blot analyses of GST-p37 fusion proteins with α-GST (C) and CA279 antibodies (D). Mp37 represents the mycoplasmal p37 protein from the extract of mycoplasma-infected cancer cells. The asterisks indicate partial degradation of GST-p37 fusion proteins.
