# Supplementary figures and images for: Mapping of a Mycoplasma-Neutralizing Epitope on the Mycoplasmal p37 Protein
Source: PLoS One. 2016 Dec 30;11(12):e0169091. doi: 10.1371/journal.pone.0169091 (PMC5201277; doi:10.1371/journal.pone.0169091)

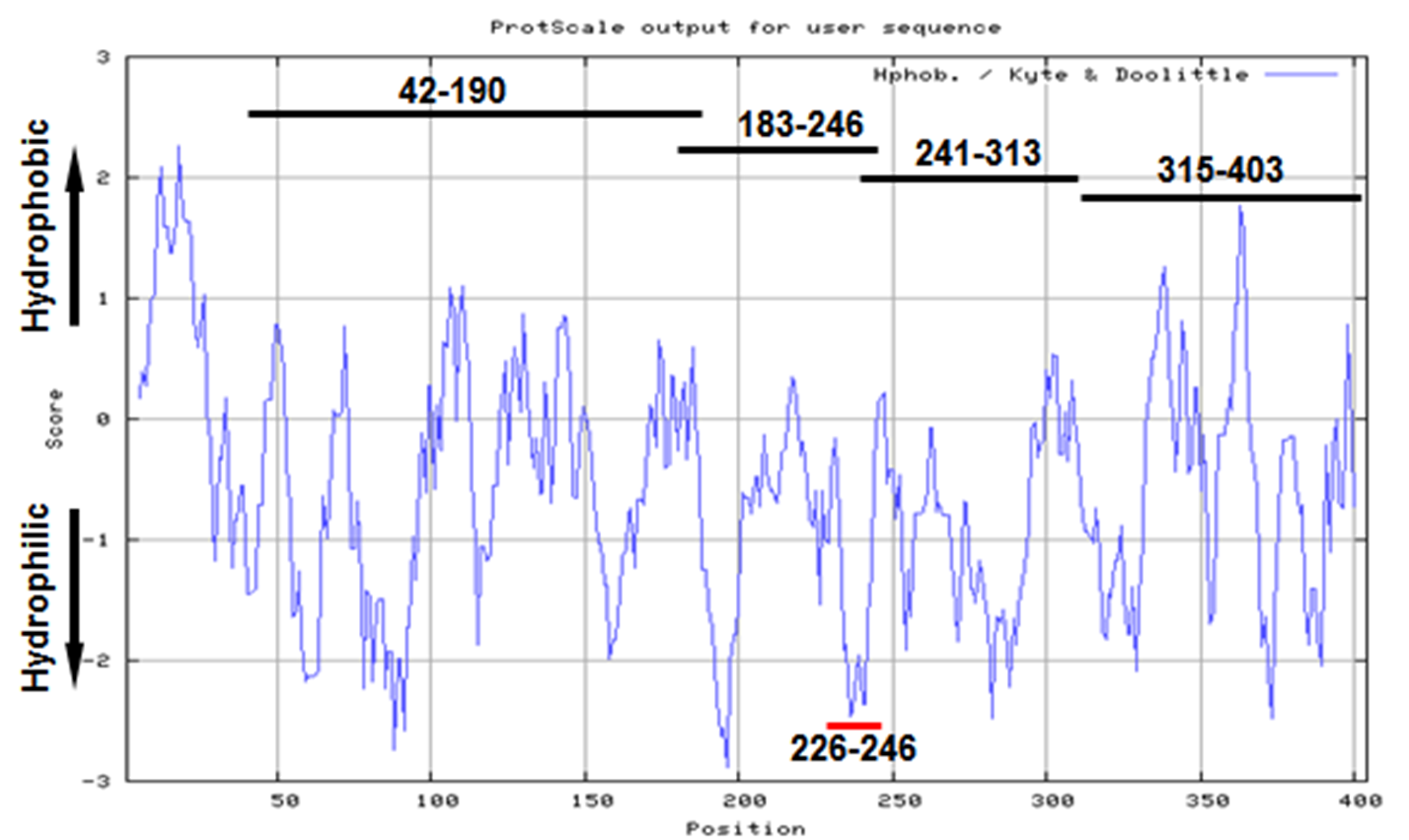

Supplement: S1 Fig — The hydrophobicity profile was obtained using ProtScale (http://www.expasy.ch/cgi-bin/protscale.pl), using a window length of 9. Hydrophobic regions are above the line, and hydrophilic regions are below. The black bars indicate the p37 DNA fragments subcloned, and the red bar indicates the epitope of CA27. (TIF) [file pone.0169091.s001.tif]

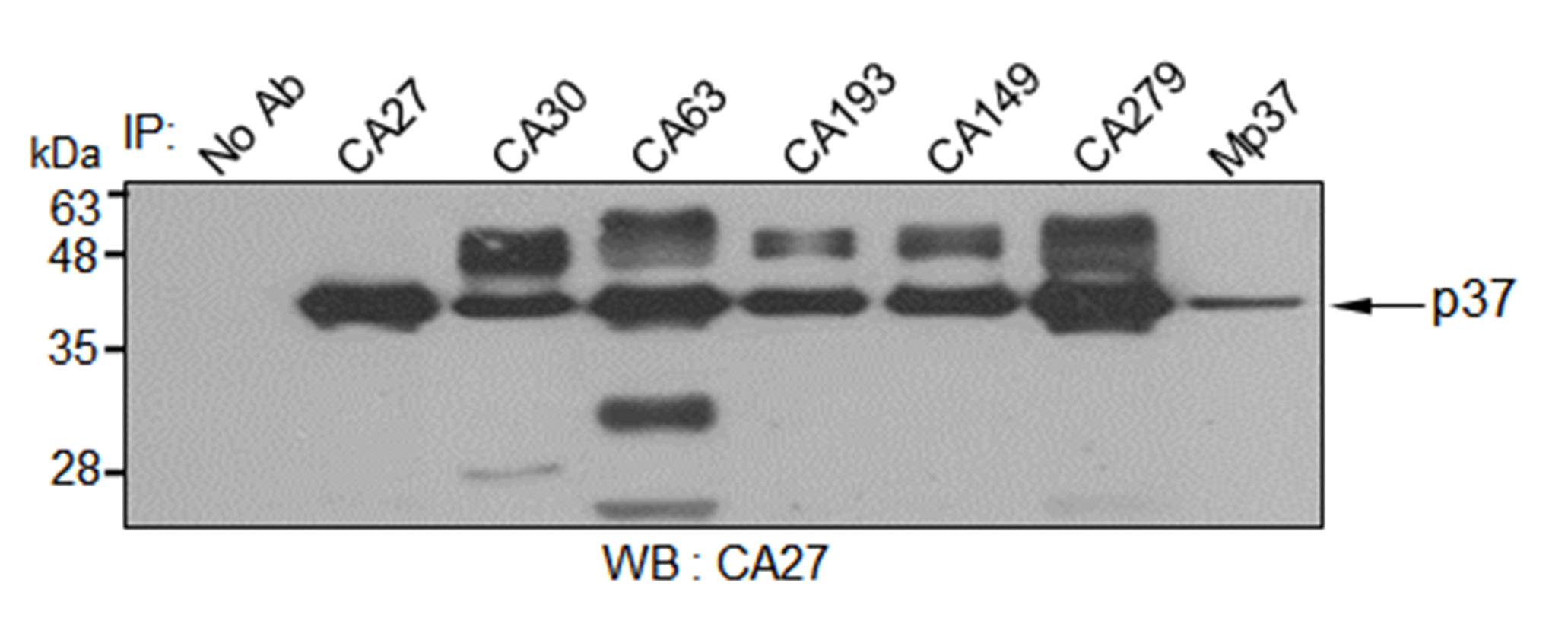

Supplement: S2 Fig — Mycoplasma-infected A549 cells were subjected to immunoprecipitation with 6 indicated antibodies. Immunoprecipitated molecules were analyzed by Western blot analysis with CA27. Mp37 represents the mycoplasmal p37 protein from the extract of mycoplasma-infected cancer cells. The arrow indicates the position of the p37 protein. (TIF) [file pone.0169091.s002.tif]
